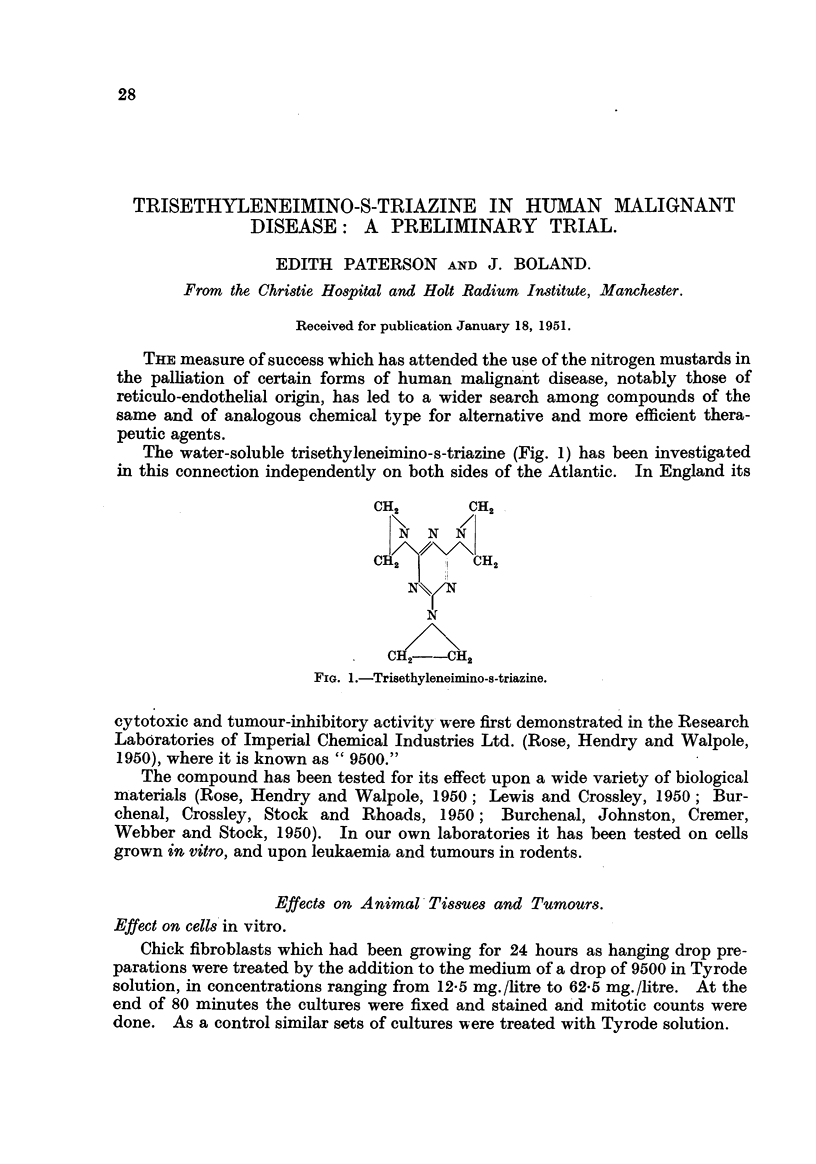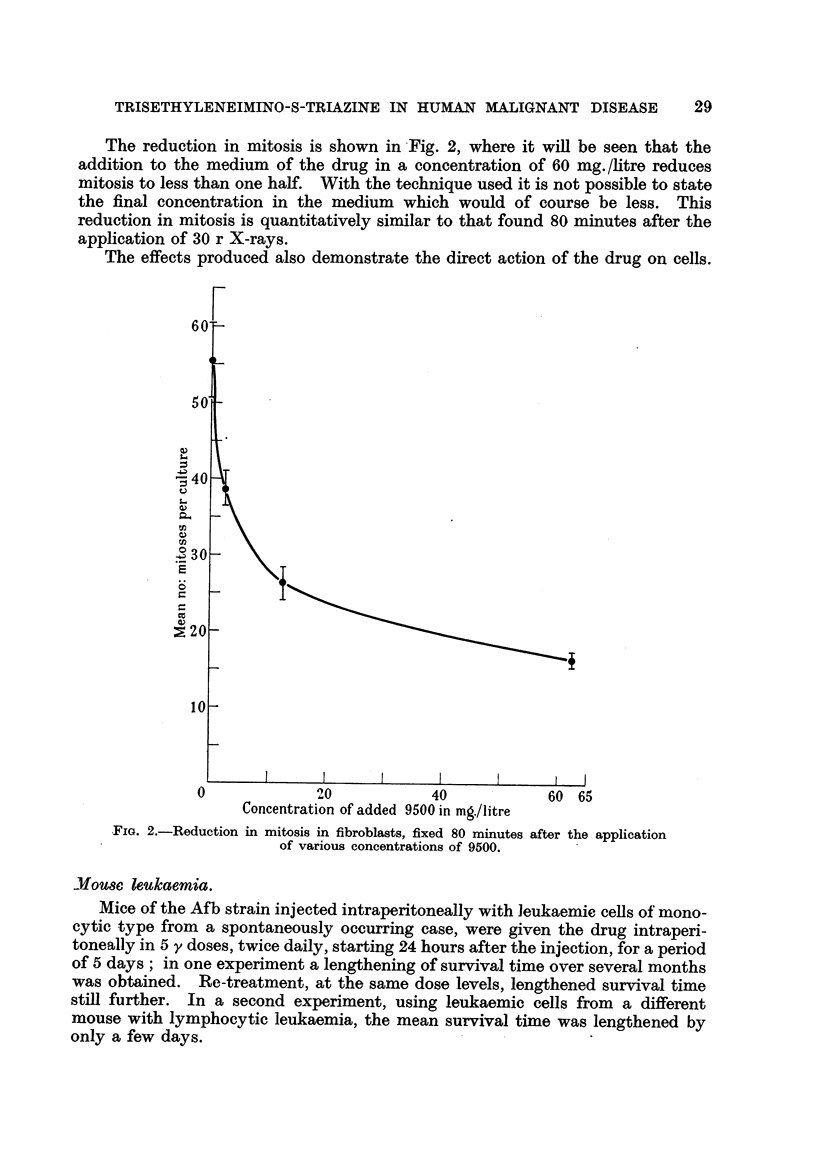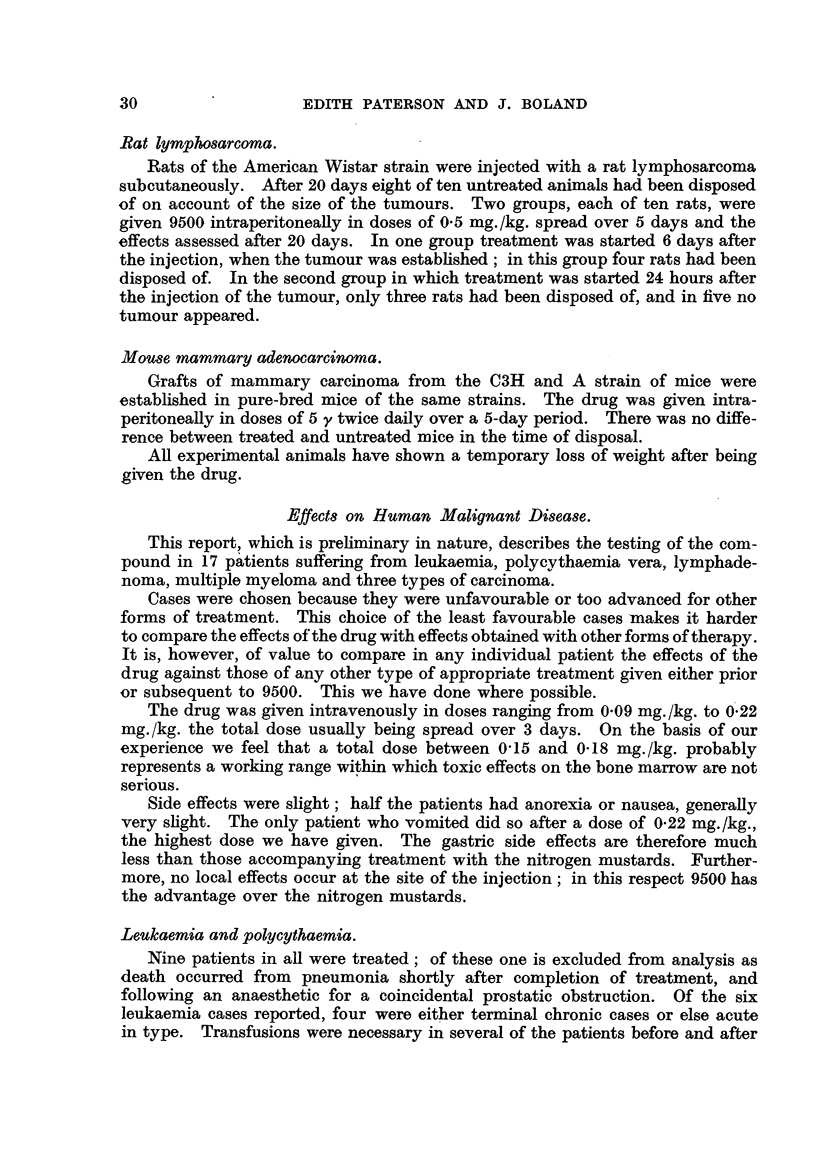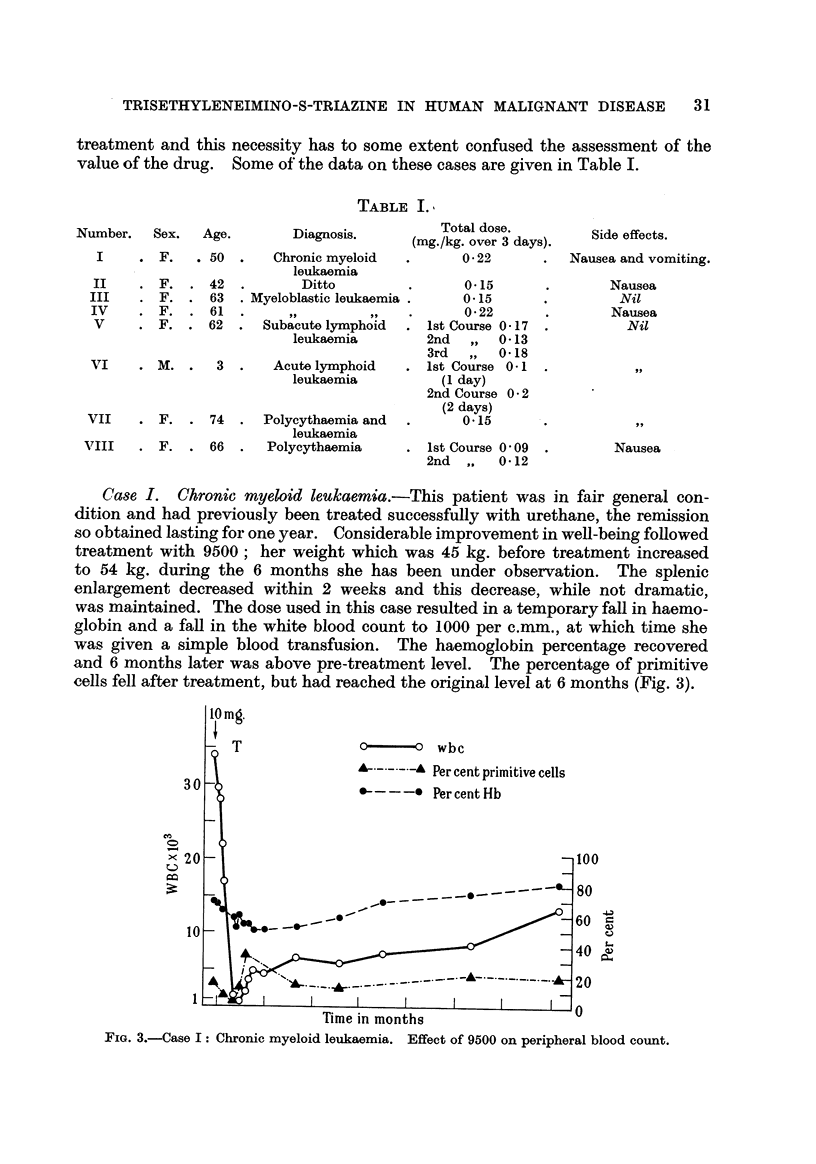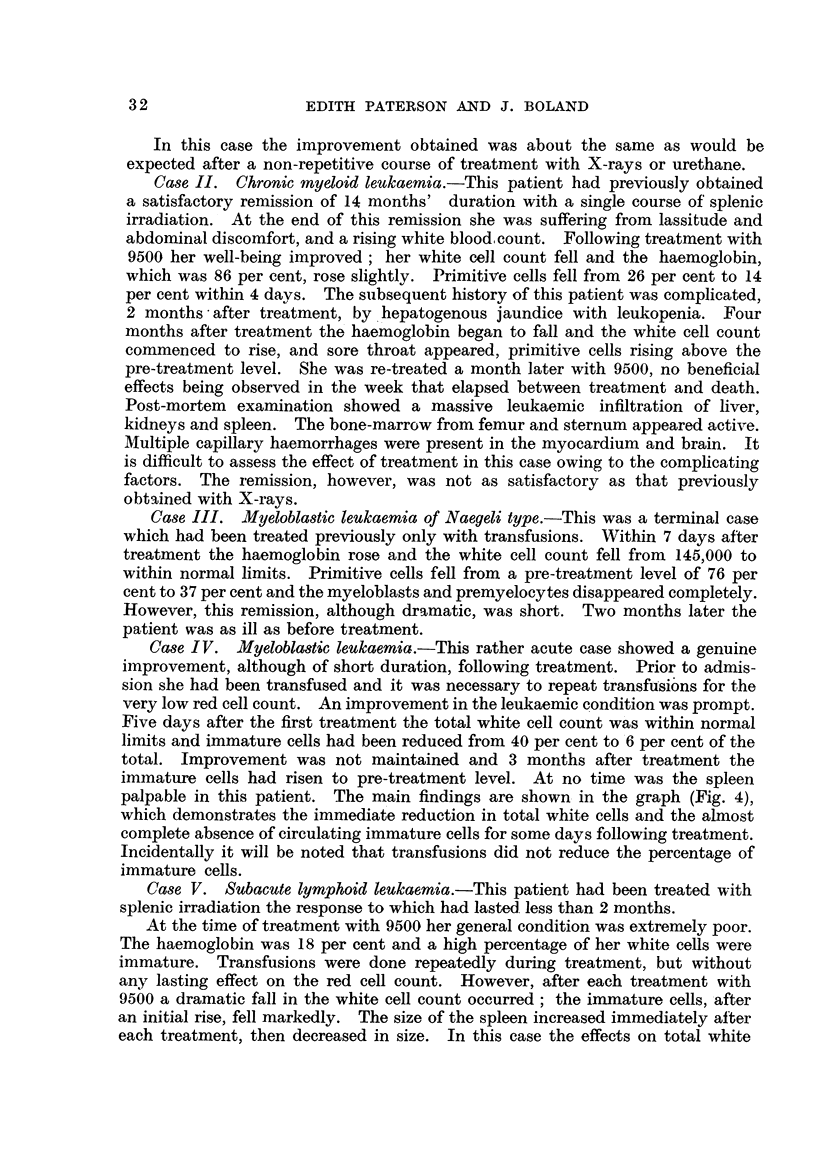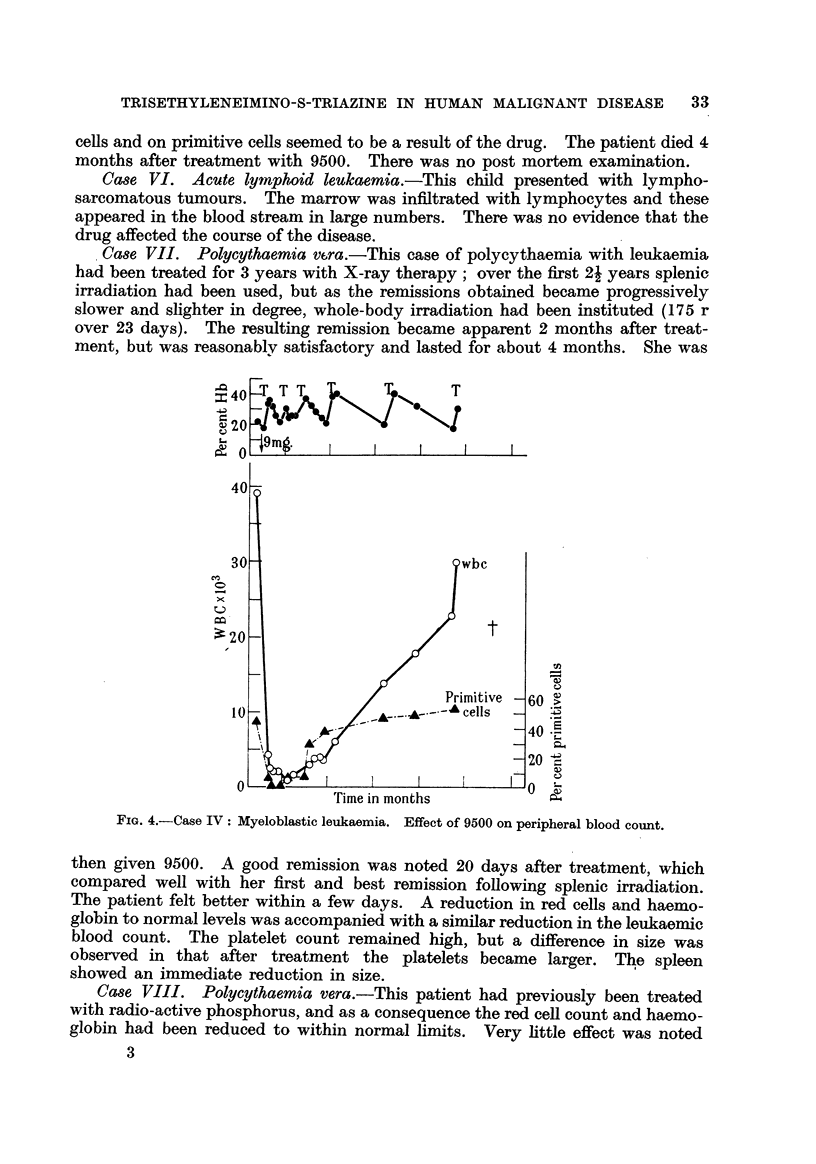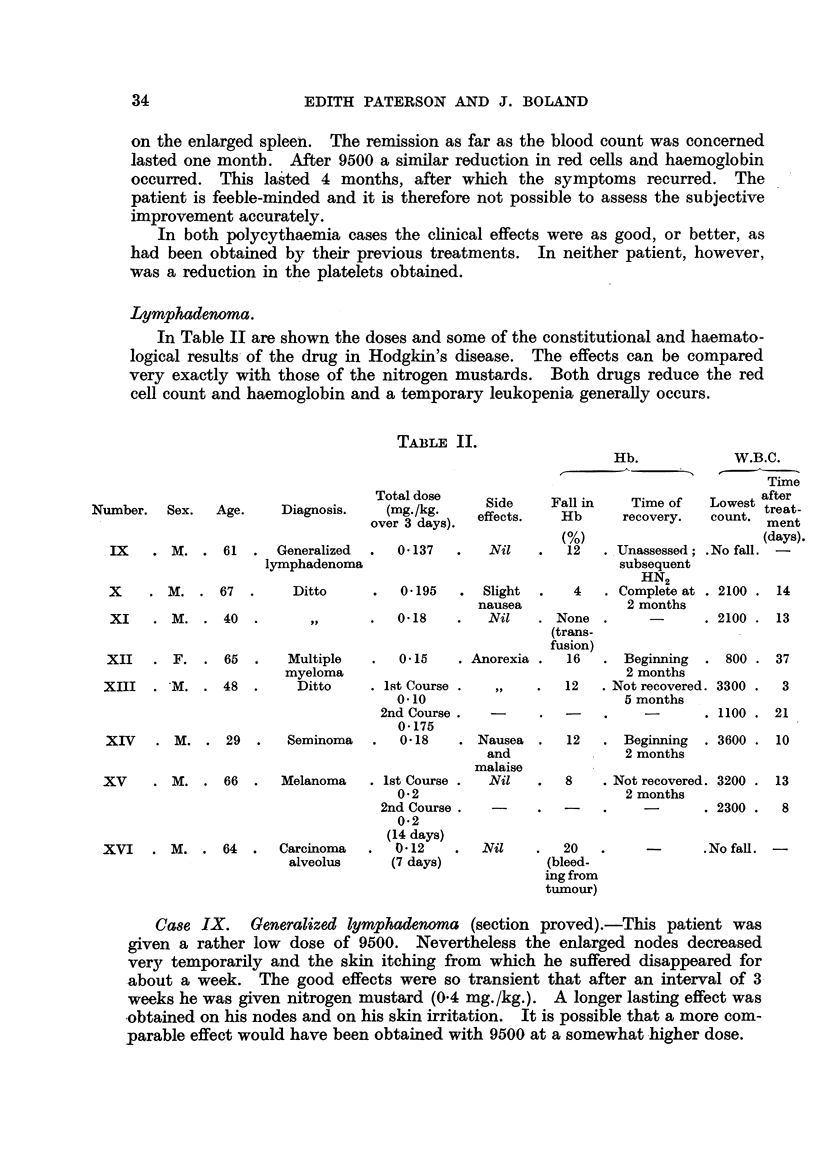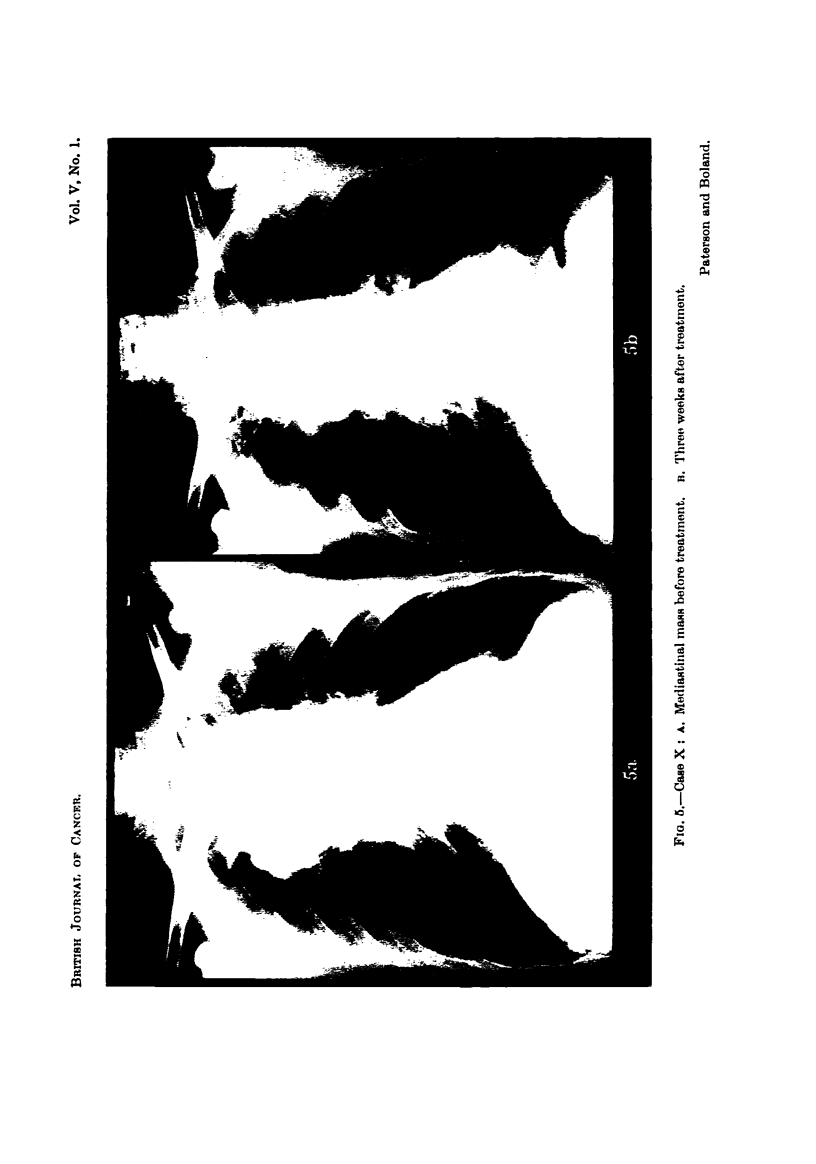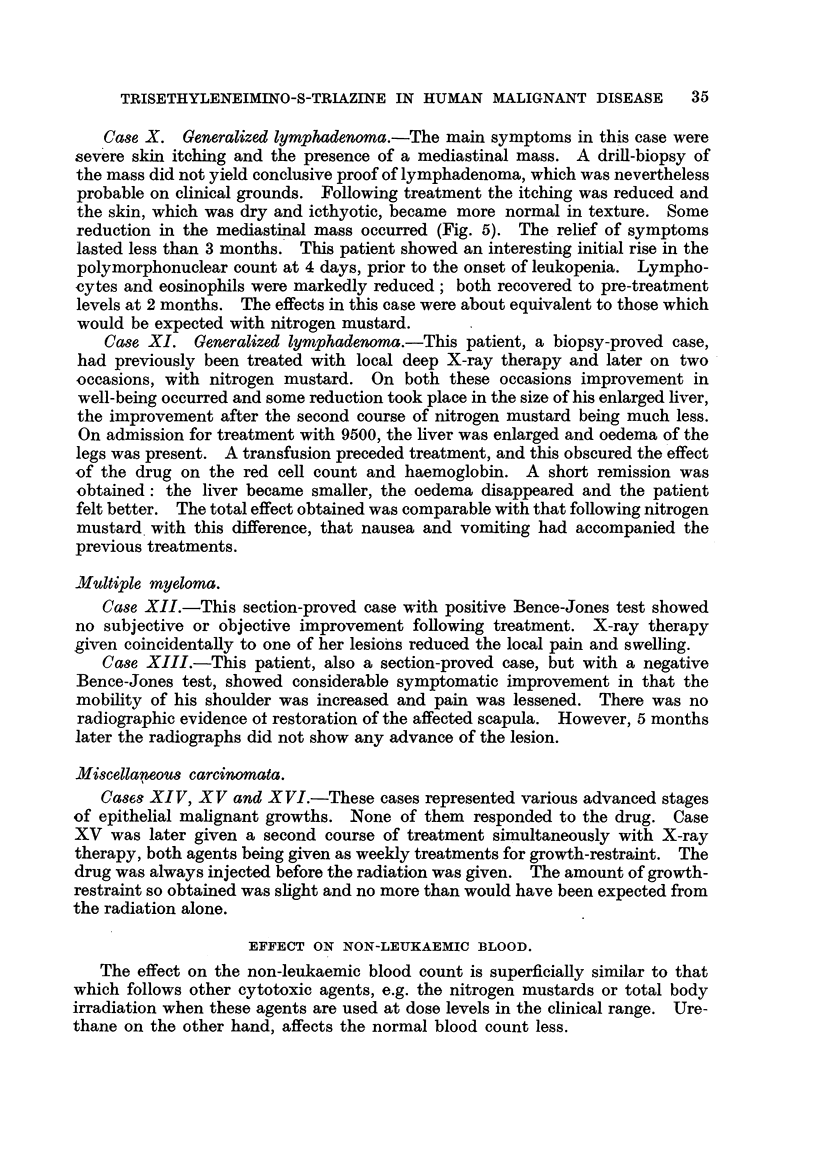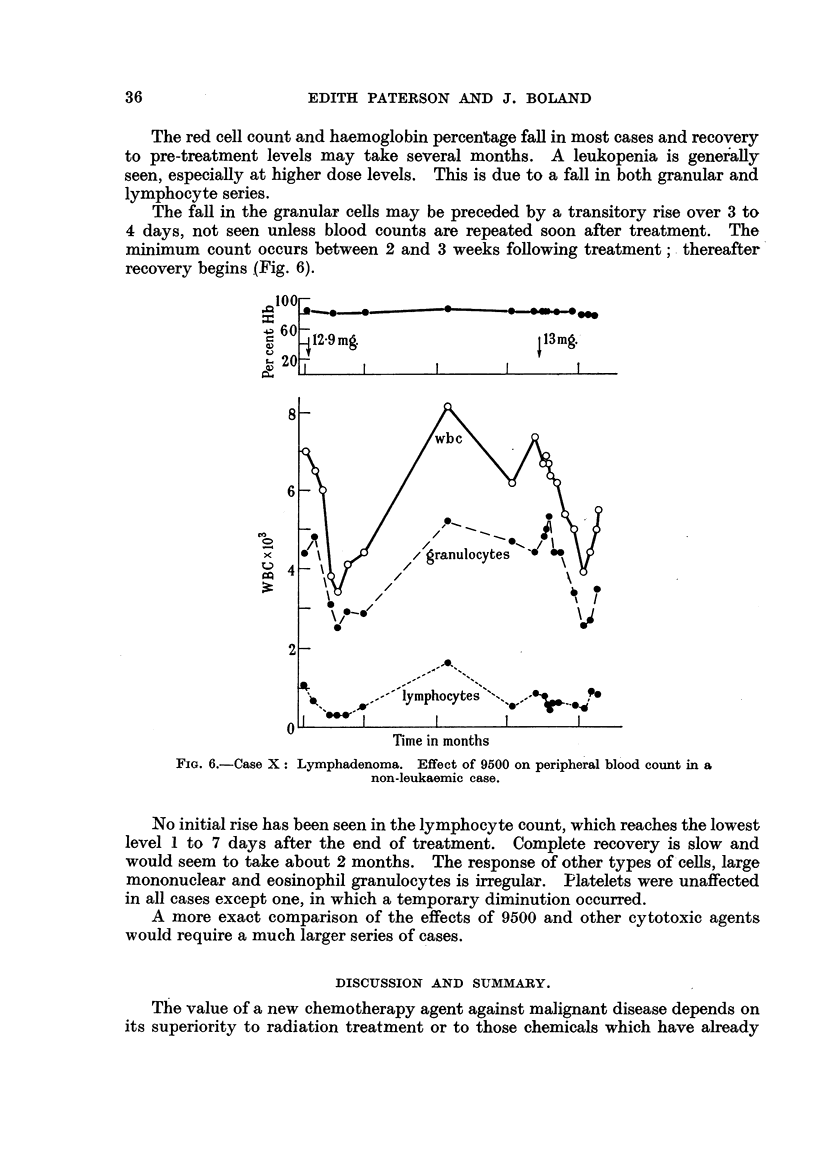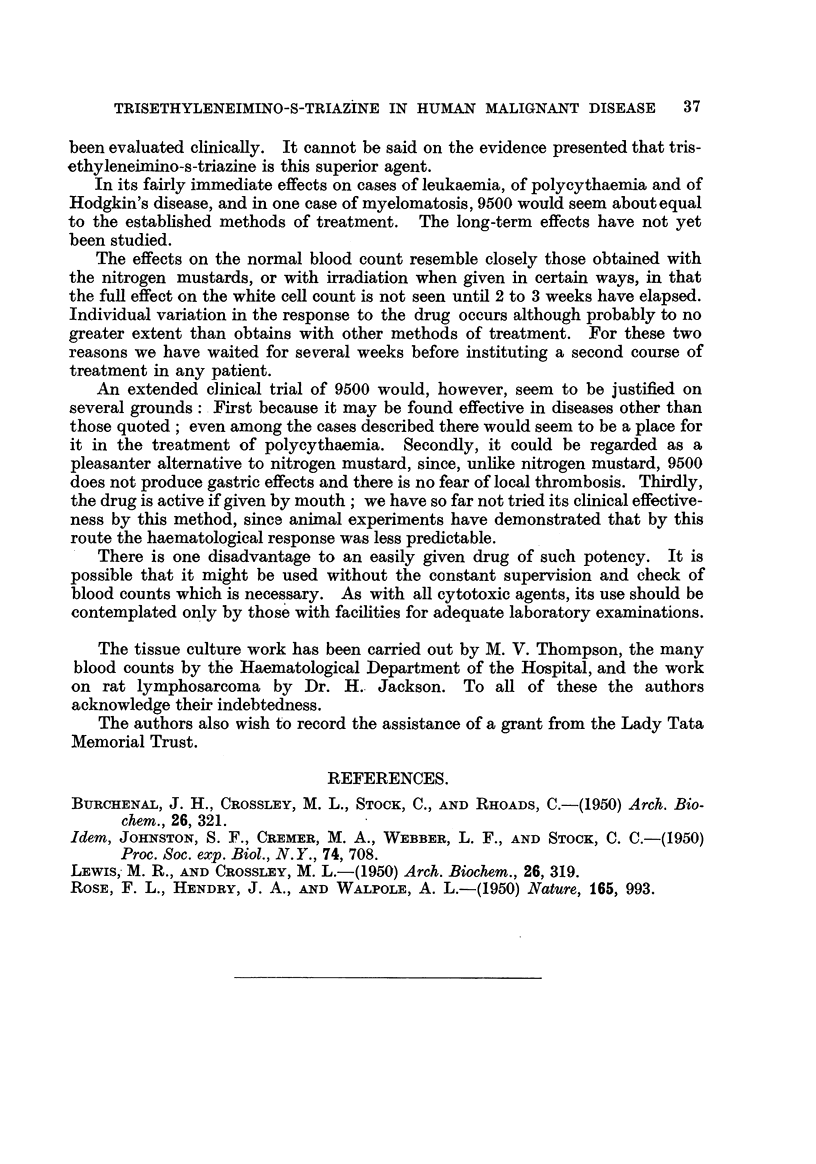# Trisethyleneimino-s-Triazine in Human Malignant Disease: A Preliminary Trial

**DOI:** 10.1038/bjc.1951.3

**Published:** 1951-03

**Authors:** Edith Paterson, J. Boland

## Abstract

**Images:**


### TRISETHYLENEIMINO-S-TRIAZINE IN HUMAN MALIGNANT DISEASE: A PRELIMINARY TRIAL.

EDITH PATERSON AND J. BOLAND.

*From the Christie Hospital and Holt Radium Institute, Manchester.*

Received for publication January 18, 1951.

THE measure of success which has attended the use of the nitrogen mustards in the palliation of certain forms of human malignant disease, notably those of reticulo-endothelial origin, has led to a wider search among compounds of the same and of analogous chemical type for alternative and more efficient therapeutic agents.

The water-soluble trisethyleneimino-s-triazine (Fig. 1) has been investigated in this connection independently on both sides of the Atlantic. In England its

FIG. 1.—Trisethyleneimino-s-triazine.

cytotoxic and tumour-inhibitory activity were first demonstrated in the Research Laboratories of Imperial Chemical Industries Ltd. (Rose, Hendry and Walpole, 1950), where it is known as " 9500."

The compound has been tested for its effect upon a wide variety of biological materials (Rose, Hendry and Walpole, 1950; Lewis and Crossley, 1950; Burchenal, Crossley, Stock and Rhoads, 1950; Burchenal, Johnston, Cremer, Webber and Stock, 1950). In our own laboratories it has been tested on cells grown *in vitro*, and upon leukaemia and tumours in rodents.

### *Effects on Animal Tissues and Tumours.*

*Effect on cells* in vitro.

Chick fibroblasts which had been growing for 24 hours as hanging drop preparations were treated by the addition to the medium of a drop of 9500 in Tyrode solution, in concentrations ranging from 12·5 mg./litre to 62·5 mg./litre. At the end of 80 minutes the cultures were fixed and stained and mitotic counts were done. As a control similar sets of cultures were treated with Tyrode solution.

The reduction in mitosis is shown in Fig. 2, where it will be seen that the addition to the medium of the drug in a concentration of 60 mg./litre reduces mitosis to less than one half. With the technique used it is not possible to state the final concentration in the medium which would of course be less. This reduction in mitosis is quantitatively similar to that found 80 minutes after the application of 30 r X-rays.

The effects produced also demonstrate the direct action of the drug on cells.

FIG. 2.—Reduction in mitosis in fibroblasts, fixed 80 minutes after the application of various concentrations of 9500.

*Mouse leukaemia.*

Mice of the Afb strain injected intraperitoneally with leukaemic cells of monocytic type from a spontaneously occurring case, were given the drug intraperitoneally in 5 $\gamma$ doses, twice daily, starting 24 hours after the injection, for a period of 5 days ; in one experiment a lengthening of survival time over several months was obtained. Re-treatment, at the same dose levels, lengthened survival time still further. In a second experiment, using leukaemic cells from a different mouse with lymphocytic leukaemia, the mean survival time was lengthened by only a few days.

*Rat lymphosarcoma.*

Rats of the American Wistar strain were injected with a rat lymphosarcoma subcutaneously. After 20 days eight of ten untreated animals had been disposed of on account of the size of the tumours. Two groups, each of ten rats, were given 9500 intraperitoneally in doses of 0·5 mg./kg. spread over 5 days and the effects assessed after 20 days. In one group treatment was started 6 days after the injection, when the tumour was established ; in this group four rats had been disposed of. In the second group in which treatment was started 24 hours after the injection of the tumour, only three rats had been disposed of, and in five no tumour appeared.

*Mouse mammary adenocarcinoma.*

Grafts of mammary carcinoma from the C3H and A strain of mice were established in pure-bred mice of the same strains. The drug was given intraperitoneally in doses of 5 $\gamma$ twice daily over a 5-day period. There was no difference between treated and untreated mice in the time of disposal.

All experimental animals have shown a temporary loss of weight after being given the drug.

### *Effects on Human Malignant Disease.*

This report, which is preliminary in nature, describes the testing of the compound in 17 patients suffering from leukaemia, polycythaemia vera, lymphadenoma, multiple myeloma and three types of carcinoma.

Cases were chosen because they were unfavourable or too advanced for other forms of treatment. This choice of the least favourable cases makes it harder to compare the effects of the drug with effects obtained with other forms of therapy. It is, however, of value to compare in any individual patient the effects of the drug against those of any other type of appropriate treatment given either prior or subsequent to 9500. This we have done where possible.

The drug was given intravenously in doses ranging from 0·09 mg./kg. to 0·22 mg./kg. the total dose usually being spread over 3 days. On the basis of our experience we feel that a total dose between 0·15 and 0·18 mg./kg. probably represents a working range within which toxic effects on the bone marrow are not serious.

Side effects were slight ; half the patients had anorexia or nausea, generally very slight. The only patient who vomited did so after a dose of 0·22 mg./kg., the highest dose we have given. The gastric side effects are therefore much less than those accompanying treatment with the nitrogen mustards. Furthermore, no local effects occur at the site of the injection ; in this respect 9500 has the advantage over the nitrogen mustards.

*Leukaemia and polycythaemia.*

Nine patients in all were treated ; of these one is excluded from analysis as death occurred from pneumonia shortly after completion of treatment, and following an anaesthetic for a coincidental prostatic obstruction. Of the six leukaemia cases reported, four were either terminal chronic cases or else acute in type. Transfusions were necessary in several of the patients before and after treatment and this necessity has to some extent confused the assessment of the value of the drug. Some of the data on these cases are given in Table I.

TABLE I.

| Number. | Sex. | Age. | Diagnosis. | Total dose. (mg./kg. over 3 days). | Side effects. |
|---|---|---|---|---|---|
| I | F. | 50 | Chronic myeloid leukaemia | 0·22 | Nausea and vomiting. |
| II | F. | 42 | Ditto | 0·15 | Nausea |
| III | F. | 63 | Myeloblastic leukaemia | 0·15 | *Nil* |
| IV | F. | 61 | ,, ,, | 0·22 | Nausea |
| V | F. | 62 | Subacute lymphoid leukaemia | 1st Course 0·17<br>2nd ,, 0·13<br>3rd ,, 0·18 | *Nil* |
| VI | M. | 3 | Acute lymphoid leukaemia | 1st Course 0·1 (1 day)<br>2nd Course 0·2 (2 days) | ,, |
| VII | F. | 74 | Polycythaemia and leukaemia | 0·15 | ,, |
| VIII | F. | 66 | Polycythaemia | 1st Course 0·09<br>2nd ,, 0·12 | Nausea |

*Case I. Chronic myeloid leukaemia.*—This patient was in fair general condition and had previously been treated successfully with urethane, the remission so obtained lasting for one year. Considerable improvement in well-being followed treatment with 9500 ; her weight which was 45 kg. before treatment increased to 54 kg. during the 6 months she has been under observation. The splenic enlargement decreased within 2 weeks and this decrease, while not dramatic, was maintained. The dose used in this case resulted in a temporary fall in haemoglobin and a fall in the white blood count to 1000 per c.mm., at which time she was given a simple blood transfusion. The haemoglobin percentage recovered and 6 months later was above pre-treatment level. The percentage of primitive cells fell after treatment, but had reached the original level at 6 months (Fig. 3).

FIG. 3.—Case I : Chronic myeloid leukaemia. Effect of 9500 on peripheral blood count.

In this case the improvement obtained was about the same as would be expected after a non-repetitive course of treatment with X-rays or urethane.

*Case II. Chronic myeloid leukaemia.*—This patient had previously obtained a satisfactory remission of 14 months' duration with a single course of splenic irradiation. At the end of this remission she was suffering from lassitude and abdominal discomfort, and a rising white blood count. Following treatment with 9500 her well-being improved ; her white cell count fell and the haemoglobin, which was 86 per cent, rose slightly. Primitive cells fell from 26 per cent to 14 per cent within 4 days. The subsequent history of this patient was complicated, 2 months after treatment, by hepatogenous jaundice with leukopenia. Four months after treatment the haemoglobin began to fall and the white cell count commenced to rise, and sore throat appeared, primitive cells rising above the pre-treatment level. She was re-treated a month later with 9500, no beneficial effects being observed in the week that elapsed between treatment and death. Post-mortem examination showed a massive leukaemic infiltration of liver, kidneys and spleen. The bone-marrow from femur and sternum appeared active. Multiple capillary haemorrhages were present in the myocardium and brain. It is difficult to assess the effect of treatment in this case owing to the complicating factors. The remission, however, was not as satisfactory as that previously obtained with X-rays.

*Case III. Myeloblastic leukaemia of Naegeli type.*—This was a terminal case which had been treated previously only with transfusions. Within 7 days after treatment the haemoglobin rose and the white cell count fell from 145,000 to within normal limits. Primitive cells fell from a pre-treatment level of 76 per cent to 37 per cent and the myeloblasts and premyelocytes disappeared completely. However, this remission, although dramatic, was short. Two months later the patient was as ill as before treatment.

*Case IV. Myeloblastic leukaemia.*—This rather acute case showed a genuine improvement, although of short duration, following treatment. Prior to admission she had been transfused and it was necessary to repeat transfusions for the very low red cell count. An improvement in the leukaemic condition was prompt. Five days after the first treatment the total white cell count was within normal limits and immature cells had been reduced from 40 per cent to 6 per cent of the total. Improvement was not maintained and 3 months after treatment the immature cells had risen to pre-treatment level. At no time was the spleen palpable in this patient. The main findings are shown in the graph (Fig. 4), which demonstrates the immediate reduction in total white cells and the almost complete absence of circulating immature cells for some days following treatment. Incidentally it will be noted that transfusions did not reduce the percentage of immature cells.

*Case V. Subacute lymphoid leukaemia.*—This patient had been treated with splenic irradiation the response to which had lasted less than 2 months.

At the time of treatment with 9500 her general condition was extremely poor. The haemoglobin was 18 per cent and a high percentage of her white cells were immature. Transfusions were done repeatedly during treatment, but without any lasting effect on the red cell count. However, after each treatment with 9500 a dramatic fall in the white cell count occurred ; the immature cells, after an initial rise, fell markedly. The size of the spleen increased immediately after each treatment, then decreased in size. In this case the effects on total white cells and on primitive cells seemed to be a result of the drug. The patient died 4 months after treatment with 9500. There was no post mortem examination.

*Case VI. Acute lymphoid leukaemia.*—This child presented with lymphosarcomatous tumours. The marrow was infiltrated with lymphocytes and these appeared in the blood stream in large numbers. There was no evidence that the drug affected the course of the disease.

*Case VII. Polycythaemia vera.*—This case of polycythaemia with leukaemia had been treated for 3 years with X-ray therapy ; over the first 2½ years splenic irradiation had been used, but as the remissions obtained became progressively slower and slighter in degree, whole-body irradiation had been instituted (175 r over 23 days). The resulting remission became apparent 2 months after treatment, but was reasonably satisfactory and lasted for about 4 months. She was

FIG. 4.—Case IV : Myeloblastic leukaemia. Effect of 9500 on peripheral blood count.

then given 9500. A good remission was noted 20 days after treatment, which compared well with her first and best remission following splenic irradiation. The patient felt better within a few days. A reduction in red cells and haemoglobin to normal levels was accompanied with a similar reduction in the leukaemic blood count. The platelet count remained high, but a difference in size was observed in that after treatment the platelets became larger. The spleen showed an immediate reduction in size.

*Case VIII. Polycythaemia vera.*—This patient had previously been treated with radio-active phosphorus, and as a consequence the red cell count and haemoglobin had been reduced to within normal limits. Very little effect was noted on the enlarged spleen. The remission as far as the blood count was concerned lasted one month. After 9500 a similar reduction in red cells and haemoglobin occurred. This lasted 4 months, after which the symptoms recurred. The patient is feeble-minded and it is therefore not possible to assess the subjective improvement accurately.

In both polycythaemia cases the clinical effects were as good, or better, as had been obtained by their previous treatments. In neither patient, however, was a reduction in the platelets obtained.

*Lymphadenoma.*

In Table II are shown the doses and some of the constitutional and haematological results of the drug in Hodgkin's disease. The effects can be compared very exactly with those of the nitrogen mustards. Both drugs reduce the red cell count and haemoglobin and a temporary leukopenia generally occurs.

TABLE II.

| Number. | Sex. | Age. | Diagnosis. | Total dose (mg./kg. over 3 days). | Side effects. | Hb. Fall in Hb (%) | Hb. Time of recovery. | W.B.C. Lowest count. | W.B.C. Time after treatment (days). |
|---|---|---|---|---|---|---|---|---|---|
| IX | M. | 61 | Generalized lymphadenoma | 0·137 | *Nil* | 12 | Unassessed; subsequent $HN_2$ | No fall. | — |
| X | M. | 67 | Ditto | 0·195 | Slight nausea | 4 | Complete at 2 months | 2100 | 14 |
| XI | M. | 40 | ,, | 0·18 | *Nil* | None (transfusion) | — | 2100 | 13 |
| XII | F. | 65 | Multiple myeloma | 0·15 | Anorexia | 16 | Beginning 2 months | 800 | 37 |
| XIII | M. | 48 | Ditto | 1st Course 0·10 | ,, | 12 | Not recovered. 5 months | 3300 | 3 |
| | | | | 2nd Course 0·175 | — | — | — | 1100 | 21 |
| XIV | M. | 29 | Seminoma | 0·18 | Nausea and malaise | 12 | Beginning 2 months | 3600 | 10 |
| XV | M. | 66 | Melanoma | 1st Course 0·2 | *Nil* | 8 | Not recovered. 2 months | 3200 | 13 |
| | | | | 2nd Course 0·2 (14 days) | — | — | — | 2300 | 8 |
| XVI | M. | 64 | Carcinoma alveolus | 0·12 (7 days) | *Nil* | 20 (bleeding from tumour) | — | No fall. | — |

*Case IX. Generalized lymphadenoma* (section proved).—This patient was given a rather low dose of 9500. Nevertheless the enlarged nodes decreased very temporarily and the skin itching from which he suffered disappeared for about a week. The good effects were so transient that after an interval of 3 weeks he was given nitrogen mustard (0·4 mg./kg.). A longer lasting effect was obtained on his nodes and on his skin irritation. It is possible that a more comparable effect would have been obtained with 9500 at a somewhat higher dose.

Fig. 5.—Case X : a. Mediastinal mass before treatment. b. Three weeks after treatment.

Paterson and Boland.

*Case X. Generalized lymphadenoma.*—The main symptoms in this case were severe skin itching and the presence of a mediastinal mass. A drill-biopsy of the mass did not yield conclusive proof of lymphadenoma, which was nevertheless probable on clinical grounds. Following treatment the itching was reduced and the skin, which was dry and icthyotic, became more normal in texture. Some reduction in the mediastinal mass occurred (Fig. 5). The relief of symptoms lasted less than 3 months. This patient showed an interesting initial rise in the polymorphonuclear count at 4 days, prior to the onset of leukopenia. Lymphocytes and eosinophils were markedly reduced ; both recovered to pre-treatment levels at 2 months. The effects in this case were about equivalent to those which would be expected with nitrogen mustard.

*Case XI. Generalized lymphadenoma.*—This patient, a biopsy-proved case, had previously been treated with local deep X-ray therapy and later on two occasions, with nitrogen mustard. On both these occasions improvement in well-being occurred and some reduction took place in the size of his enlarged liver, the improvement after the second course of nitrogen mustard being much less. On admission for treatment with 9500, the liver was enlarged and oedema of the legs was present. A transfusion preceded treatment, and this obscured the effect of the drug on the red cell count and haemoglobin. A short remission was obtained : the liver became smaller, the oedema disappeared and the patient felt better. The total effect obtained was comparable with that following nitrogen mustard with this difference, that nausea and vomiting had accompanied the previous treatments.

*Multiple myeloma.*

*Case XII.*—This section-proved case with positive Bence-Jones test showed no subjective or objective improvement following treatment. X-ray therapy given coincidentally to one of her lesions reduced the local pain and swelling.

*Case XIII.*—This patient, also a section-proved case, but with a negative Bence-Jones test, showed considerable symptomatic improvement in that the mobility of his shoulder was increased and pain was lessened. There was no radiographic evidence ot restoration of the affected scapula. However, 5 months later the radiographs did not show any advance of the lesion.

*Miscellaneous carcinomata.*

*Cases XIV, XV and XVI.*—These cases represented various advanced stages of epithelial malignant growths. None of them responded to the drug. Case XV was later given a second course of treatment simultaneously with X-ray therapy, both agents being given as weekly treatments for growth-restraint. The drug was always injected before the radiation was given. The amount of growth-restraint so obtained was slight and no more than would have been expected from the radiation alone.

### EFFECT ON NON-LEUKAEMIC BLOOD.

The effect on the non-leukaemic blood count is superficially similar to that which follows other cytotoxic agents, e.g. the nitrogen mustards or total body irradiation when these agents are used at dose levels in the clinical range. Urethane on the other hand, affects the normal blood count less.

The red cell count and haemoglobin percentage fall in most cases and recovery to pre-treatment levels may take several months. A leukopenia is generally seen, especially at higher dose levels. This is due to a fall in both granular and lymphocyte series.

The fall in the granular cells may be preceded by a transitory rise over 3 to 4 days, not seen unless blood counts are repeated soon after treatment. The minimum count occurs between 2 and 3 weeks following treatment; thereafter recovery begins (Fig. 6).

FIG. 6.—Case X: Lymphadenoma. Effect of 9500 on peripheral blood count in a non-leukaemic case.

No initial rise has been seen in the lymphocyte count, which reaches the lowest level 1 to 7 days after the end of treatment. Complete recovery is slow and would seem to take about 2 months. The response of other types of cells, large mononuclear and eosinophil granulocytes is irregular. Platelets were unaffected in all cases except one, in which a temporary diminution occurred.

A more exact comparison of the effects of 9500 and other cytotoxic agents would require a much larger series of cases.

DISCUSSION AND SUMMARY.

The value of a new chemotherapy agent against malignant disease depends on its superiority to radiation treatment or to those chemicals which have already been evaluated clinically. It cannot be said on the evidence presented that tris-ethyleneimino-s-triazine is this superior agent.

In its fairly immediate effects on cases of leukaemia, of polycythaemia and of Hodgkin's disease, and in one case of myelomatosis, 9500 would seem about equal to the established methods of treatment. The long-term effects have not yet been studied.

The effects on the normal blood count resemble closely those obtained with the nitrogen mustards, or with irradiation when given in certain ways, in that the full effect on the white cell count is not seen until 2 to 3 weeks have elapsed. Individual variation in the response to the drug occurs although probably to no greater extent than obtains with other methods of treatment. For these two reasons we have waited for several weeks before instituting a second course of treatment in any patient.

An extended clinical trial of 9500 would, however, seem to be justified on several grounds : First because it may be found effective in diseases other than those quoted ; even among the cases described there would seem to be a place for it in the treatment of polycythaemia. Secondly, it could be regarded as a pleasanter alternative to nitrogen mustard, since, unlike nitrogen mustard, 9500 does not produce gastric effects and there is no fear of local thrombosis. Thirdly, the drug is active if given by mouth ; we have so far not tried its clinical effectiveness by this method, since animal experiments have demonstrated that by this route the haematological response was less predictable.

There is one disadvantage to an easily given drug of such potency. It is possible that it might be used without the constant supervision and check of blood counts which is necessary. As with all cytotoxic agents, its use should be contemplated only by those with facilities for adequate laboratory examinations.

The tissue culture work has been carried out by M. V. Thompson, the many blood counts by the Haematological Department of the Hospital, and the work on rat lymphosarcoma by Dr. H. Jackson. To all of these the authors acknowledge their indebtedness.

The authors also wish to record the assistance of a grant from the Lady Tata Memorial Trust.

### REFERENCES.

BURCHENAL, J. H., CROSSLEY, M. L., STOCK, C., AND RHOADS, C.—(1950) *Arch. Biochem.*, **26**, 321.

*Idem*, JOHNSTON, S. F., CREMER, M. A., WEBBER, L. F., AND STOCK, C. C.—(1950) *Proc. Soc. exp. Biol.*, *N.Y.*, **74**, 708.

LEWIS, M. R., AND CROSSLEY, M. L.—(1950) *Arch. Biochem.*, **26**, 319.

ROSE, F. L., HENDRY, J. A., AND WALPOLE, A. L.—(1950) *Nature*, **165**, 993.